# Changes in cause-specific mortality trends across occupations in working-age Japanese women from 1980 to 2015: a cross-sectional analysis

**DOI:** 10.1186/s12905-022-01621-4

**Published:** 2022-02-22

**Authors:** Bibha Dhungel, Kuniyasu Takagi, Shijan Acharya, Koji Wada, Stuart Gilmour

**Affiliations:** 1 Graduate School of Public Health, St. Luke’s International University, Tsukiji, Tokyo, Japan; 2 Department of Health Policy, National Centre for Child Health and Development, Setagaya, Tokyo, Japan; 3 Graduate School of Medicine, International University of Health and Welfare, Akasaka, Tokyo, Japan; 4 Community Empowerment for Health Promotion Programme, Nepal Red Cross Society, Nepalgunj, Nepal

**Keywords:** Mortality, Japan, Inequality, Occupation, Working age, Women, Cancer, Cerebrovascular disease, Ischaemic heart disease, Suicide

## Abstract

**Background:**

Reducing health inequalities is an important public health challenge. Many studies have examined the widening health gap by occupational class among men, but few among women. We therefore estimated variation in absolute and relative mortality by occupational category across four leading causes of mortality—cancer, ischaemic heart disease, cerebrovascular disease, and suicide—to explore how occupational class is associated with health among working women aged 25–64 in Japan.

**Methods:**

We conducted a repeated cross-sectional study using Poisson regression analysis on each five-yearly mortality data from 1980 to 2015, obtained from the National Vital Statistics and the Japanese Population Census.

**Results:**

There was a decreasing trend in mortality from all cancers, ischaemic heart disease, cerebrovascular disease, and suicide among women in all occupational groups from 1980 to 2015. Agriculture workers had higher risk of mortality than professional workers for all four causes of death. The absolute difference in mortality rates for all cancers and cerebrovascular disease was higher in 2000–2015 than 1980–1995. The mortality trend among clerks and sales workers decreased after 2000, except for suicide.

**Conclusions:**

Mortality rates from all four causes are higher among agriculture workers compared to professional workers, and attention is needed to reduce this mortality gap. Continuous monitoring of ongoing mortality trends is essential to ensure better health and wellbeing in Japan.

**Supplementary Information:**

The online version contains supplementary material available at 10.1186/s12905-022-01621-4.

## Background

Reducing health inequalities is a critical task in public health and imposes an enormous challenge. Addressing the social predictors of health is crucial. The Marmot Review, among many other previous studies, showed increasing inequalities between occupational classes [1]. Socio-economic factors such as occupation are known to affect health outcomes in life [2–4]. Recent findings also suggest that economic crises have been associated with a widening health gap by occupational class among women [5–7]. Relative measures are widely used to measure reduction in mortality. A reduction in both absolute and relative risk is important [8], but a decline in absolute mortality is essential to eliminate excess mortality [9]. However, few studies have examined how occupational class is associated with health among women in Japan [10] and how mortality inequality differs by the leading causes of death.

Japan had a considerable improvement in population health after the mid-1990s and currently has one of the best population health systems in the world [11]. However, there has been an increase in premature all-cause adult mortality, resulting in the increase in relative health inequality across occupations [12, 13]. Previous studies suggest that the inverse association between socio-economic status and mortality was lost after the financial crises in the late 1990s when there was a very large increase in mortality among professional/management workers[7]. However, these studies focused mainly on working-aged men. In 2015, Japanese women constituted 43% of the total labour force and their unemployment rate was 3.1% [14]. The number of women in the workforce is increasing and women aged 20–64 years currently constitute about 70% of female workers. However, around 44% of the employed Japanese women are engaged in part-time or temporary work compared to only 11% of employed men [14]. We therefore believe that mortality inequalities among Japanese women may be different from those among Japanese men or women in other parts of the world. In this study, we estimated the variation in absolute and relative mortality across various occupational categories and diseases and explored the inequalities in cause-specific mortality between occupational categories.

## Methods

We conducted a repeated cross-sectional study on each five-yearly mortality data, from 1980 to 2015. We obtained the mortality data from the National Vital Statistics [15] along with occupation-specific population data from the Japanese Population Census [16] conducted in the same year. The databases were merged using populations by occupation and age group from the census with deaths data by occupation and age group from the vital statistics data. We obtained permission from the Ministry of Health, Labour and Welfare of Japan for secondary use of the data. Information on occupation for the deceased was acquired from the death certificate, which contains the deceased person’s occupation before death, as given by a family member. Occupations were categorised following the Japan Standard Occupational Classification [17], as professionals (professional and technical workers), clerks, sales workers, service workers, agricultural workers (agricultural, forestry, fishery workers), manufacturing workers, managers (managers and official), transport workers and security workers. The proportion of female workers in the managers, transport, and security categories was very low, so we combined them and analysed them as ‘Other’.

The quality of data from the Japanese Vital Registration system is high with less than 10% of ill-defined codes on the registrations [18]. We excluded 1.3% of the ill-defined codes in the current dataset from the analysis. The causes of death were classified using the International Classification of Disease ninth revision for the years 1980, 1985 and 1990 and tenth revision for the years 1995, 2000, 2010 and 2015. We analysed the four leading causes of death––cancer, ischaemic heart disease, cerebrovascular disease, and suicide–among 25 to 64-year-old Japanese women [19].

### Analysis

We analysed data from female workers aged 25–64 years. We excluded workers aged < 25 years because this includes students, and those aged > 64 years because this is the age of retirement in Japan. Following standard practice for comparing rates across years, we standardised the data using the 1985 standard population of Japan in 5-year age intervals. We computed age-standardised rates for each five years from 1980 to 2015 across occupations to analyse the patterns in trends of cause-specific mortality rates.

We used a Poisson regression analysis to measure the relative mortality inequality across occupations separately by cause of death. The estimated mortality rate ratios were adjusted for time (year), age category, occupation, and a step variable. The step variable was used to indicate whether the death occurred on or before 1995, or on 2000 or after reflecting the potential change of the economic crisis in the mid-1990s across occupational categories. The first year in the data series was set as 0, while following years as 5, 10 up to 35 corresponding to the year 2015. An interaction between mortality and the step variable was also included in the model. A linear combination of the interaction variable and trend was used to compare the occupation-specific mortality rate on or before 1995, and 2000 or after. We used Stata IC version 15.1 for the analysis.

#### Analysis of mortality rates

Poisson regression model was used with population by occupation as an offset. We expressed the fundamental distribution of data as

$$y_{i} \sim Poisson(\mu_{i} )$$

where $y_{i}$ is the number of deaths from specific cause occurring at rate $\mu_{i}$ in population $n_{i}$.

$\mu_{i}$ is related to the covariates through a two-way interaction Poisson regression model as follows

$$ln\left({\mu_{i}} \right) = \alpha + ln \left( {n_{i}} \right) + \beta _{1} x_{i1} + \beta _{2} x_{i2} + \beta_{3} x_{i3} + \beta_{4} x_{i4} + \beta_{5} x_{i1} x_{i3}$$

where α is the intercept term; $x_{i1}$ is the year (0 = 1980, 5 = 1985, 10 = 1990, 15 = 1995, 20 = 2000, 25 = 2005, 30 = 2010, 35 = 2015); $x_{i2}$ is the age category (1 = 25–34, 2 = 35–44, 3 = 45–54, 4 = 55–64); $x_{i3}$ is the occupational category (1 = professional, 2 = clerk, 3 = sales, 4 = service, 5 = agriculture, 6 = manufacturing, 7 = others); and $x_{i4}$ is the step variable (0 for years 1980, 1985, 1990 and 1995, 1 for years 2000, 2005, 2010, 2015).

## Results

Table 1 shows the proportion of female workers in each occupation from 1980 to 2015. The proportion of women in professional, clerical and service categories increased over time, and those in sales, agriculture, and manufacturing decreased. The smallest groups of women workers were managers, and transport and security workers.

**Table 1:** Number and proportion of female workers aged 25–64 by occupation from 1980 to 2015

Occupational category/Year	1980	1985	1990	1995	2000	2005	2010	2015
	n (%)	n (%)	n (%)	n (%)	n (%)	n (%)	n (%)	n (%)
Professional	1,507,610 (9.0)	1,891,400 (10.5)	2,250,231 (11.8)	2,684,971 (13.5)	3,094,599 (15.1)	3,459,894 (16.7)	3,580,129 (18.0)	3,953,581 (19.9)
Clerk	3,369,822 (20.1)	4,248,922 (23.6)	5,155,485 (27.0)	5,748,954 (28.9)	6,289,031 (30.7)	6,422,961 (31.0)	5,849,554 (29.4)	5,931,116 (29.9)
Sales	2,586,857 (15.4)	2,447,212 (13.6)	2,534,197 (13.3)	2,702,863 (13.6)	2,618,387 (12.8)	2,561,132 (12.4)	2,600,469 (13.1)	2,482,037 (12.5)
Service	2,106,305 (12.6)	2,173,931 (12.1)	2,263,285 (11.8)	2,516,848 (12.7)	2,825,178 (13.8)	3,207,147 (15.5)	3,610,654 (18.2)	3,512,739 (17.7)
Agriculture	2,471,427 (14.7)	2,029,368 (11.3)	1,478,304 (7.7)	1,055,672 (5.3)	755,524 (3.7)	600,419 (2.9)	442,060 (2.2)	367,011 (1.9)
Manufacturing	4,456,927 (26.6)	4,911,261 (27.3)	5,158,278 (27.0)	4,862,147 (24.5)	4,664,292 (22.7)	4,228,532 (20.4)	3,563,491 (17.9)	3,346,382 (16.9)
Others	273,332 (1.16)	280,377 (1.6)	285,498 (1.4)	318,119 (1.5)	272,623 (1.2)	251,835 (1.1)	229,540 (1.2)	253,059 (1.2)

**Fig. 1:**
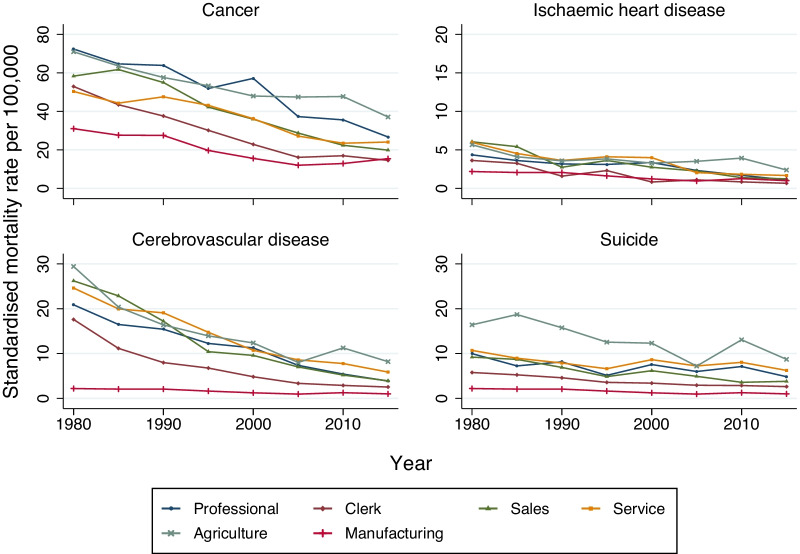
Trends in age-standardised mortality rates per 100,000 by occupation separately by cause of death

Figure 1 shows the cause-specific trends in mortality rates per 100,000 among female workers. Cancer and cerebrovascular mortality rates decreased steadily across all occupational categories until mid 2000s. However, mortality rates from ischaemic heart disease and suicide have increased in some occupational categories in recent years.

Table 2 shows the absolute and relative difference in age-standardised cause-specific mortality rates across occupations for 1980–1995 and 2000–2015. In 2015, the highest rates in mortality for all four causes of death was among agricultural workers, while clerical workers had the lowest rate in mortality. We found decreasing mortality rates by cause among women in all occupational categories from 1980 and 2015. The absolute difference in mortality from all four causes of death was larger during 1980–1995 than 2000–2015 in nearly all occupational categories. However, the relative change in mortality for all cancer, ischaemic heart disease and cerebrovascular disease was larger during 2000–2015 than 1980–1995 among professional, sales and service workers. Mortality rates from all four causes of death were highest among agricultural workers in 2015. Professional workers had the largest absolute and relative difference in mortality between 2000 and 2015 for all cancer, ischaemic heart disease and cerebrovascular disease. Between 1985 and 2015, clerks had the highest relative decrease in mortality rates from all cancers, ischaemic heart disease, and cerebrovascular disease. However, reduction in absolute mortality was much less among clerks and manufacturing workers from 1980 to 2015 for all causes of mortality.

**Table 2:** Absolute and relative difference in mortality rates for 1980–1995, and 2000–2015 across occupations

Cause of death and occupational category	Age-standardised mortality rates per 100,000				Absolute Difference^a^			Percentage change (%)^b^		
	1980	1995	2000	2015	1980–1995	2000–2015	1980–2015	1980–1995	2000–2015	1980–2015
*All cancers*										
Professional	72.4	51.9	57.1	26.7	− 20	− 30	− 46	− 28	− 53	− 63
Clerk	52.9	30.2	22.8	14.5	− 23	− 8	− 38	− 43	− 35	− 73
Sales	58.4	42.2	35.9	19.9	− 16	− 16	− 39	− 27	− 45	− 66
Service	50.4	43.1	36.2	24.1	− 7	− 12	− 26	− 14	− 33	− 52
Agriculture	71.0	53.3	48.0	37.1	− 18	− 11	− 34	− 25	− 23	− 48
Manufacturing	31.0	19.7	15.6	15.4	− 11	0	− 16	− 35	0	− 50
*Ischaemic heart disease*										
Professional	4.4	3.1	3.4	1.1	− 1	− 2	− 3	− 23	− 59	− 75
Clerk	3.6	2.3	0.8	0.7	− 1	0	− 3	− 28	0	− 81
Sales	6.0	3.6	2.7	1.2	− 2	− 1	− 5	− 33	− 37	− 80
Service	6.0	4.1	4.0	1.7	− 2	− 2	− 4	− 33	− 50	− 72
Agriculture	5.7	3.8	3.3	2.4	− 2	− 1	− 3	− 35	− 30	− 58
Manufacturing	2.2	1.6	1.2	1.0	− 1	0	− 1	− 45	0	− 55
*Cerebrovascular disease*										
Professional	20.9	12.2	11.3	3.9	− 9	− 7	− 17	− 43	− 62	− 81
Clerk	17.6	6.8	4.8	2.5	− 11	− 2	− 15	− 62	− 42	− 86
Sales	26.2	10.4	9.6	3.9	− 16	− 6	− 22	− 61	− 62	− 85
Service	24.6	14.7	10.7	5.9	− 10	− 5	− 19	− 41	− 47	− 76
Agriculture	29.4	13.9	12.4	8.2	− 15	− 4	− 21	− 51	− 32	− 72
Manufacturing	11.8	6.6	4.4	3.4	− 5	− 1	− 8	− 42	− 23	− 71
*Suicide*										
Professional	10.0	5.1	7.5	4.8	− 5	− 3	− 5	− 50	− 40	− 52
Clerk	5.8	3.6	3.4	2.6	− 2	− 1	− 3	− 34	− 29	− 55
Sales	9.2	4.8	6.2	3.8	− 4	− 2	− 5	− 43	− 32	− 59
Service	10.7	6.6	8.6	6.2	− 4	− 2	− 5	− 37	− 23	− 42
Agriculture	16.4	12.5	12.3	8.7	− 4	− 4	− 8	− 24	− 33	− 47
Manufacturing	5.1	2.9	3.0	3.3	− 2	0	− 2	− 39	0	− 35

^a^Absolute difference in age-standardised mortality rates per 100,000 between 1980 and 2015; ^b^Difference expressed as a percentage of rates in 1980 or 2000

Table 3 shows the risk of mortality across occupational categories obtained from Poisson regression analysis. Rate ratio for mortality among agriculture workers for all four causes of death is higher than among professionals. Risk of mortality for service workers is higher than professionals for ischaemic heart disease, cerebrovascular disease, and suicide, while for cancer, the risk ratio is 0.78 (95% Confidence Interval, 0.75–0.81). For clerical workers and manufacturing workers, the risk of mortality is lower than among professionals. Full result of the Poisson regression analysis is shown in Table A1 [see Additional file 1].

**Table 3:** Mortality inequalities across occupation among female workers aged 25–64 in Japan

Cause of death and occupational category	Rate ratio^a^	95% confidence interval	*p *value
*All cancers*			
Professional	Reference		
Clerk	0.50	(0.48–0.52)	< 0.01
Sales	0.76	(0.73–0.80)	< 0.01
Service	0.78	(0.75–0.81)	< 0.01
Agriculture	1.26	(1.19–1.34)	< 0.01
Manufacturing	0.38	(0.36–0.40)	< 0.01
Other	4.86	(4.61–5.11)	< 0.01
*Ischaemic heart disease*			
Professional	Reference		
Clerk	0.46	(0.37–0.55)	< 0.01
Sales	1.01	(0.81–1.20)	0.947
Service	1.20	(0.99–1.40)	0.037
Agriculture	1.70	(1.33–2.07)	< 0.01
Manufacturing	0.54	(0.43–0.64)	< 0.01
Other	5.79	(4.51–7.07)	< 0.01
*Cerebrovascular disease*			
Professional	Reference		
Clerk	0.56	(0.50–0.61)	< 0.01
Sales	1.02	(0.92–1.13)	0.671
Service	1.31	(1.19–1.43)	< 0.01
Agriculture	1.52	(1.33–1.71)	< 0.01
Manufacturing	0.54	(0.49–0.60)	< 0.01
Other	4.41	(3.85–4.98)	< 0.01
*Suicide*			
Professional	Reference		
Clerk	0.50	(0.45–0.55)	< 0.01
Sales	0.77	(0.68–0.86)	< 0.01
Service	1.20	(1.09–1.32)	< 0.01
Agriculture	1.97	(1.70–2.25)	< 0.01
Manufacturing	0.49	(0.44–0.55)	< 0.01
Other	4.83	(4.18–5.49)	< 0.01

^a^Rate ratio obtained from Poisson regression analysis adjusting for year, age category, step variable (mortality before or after 2000), occupation and interaction of step variable and occupation

Table 4 shows the rate ratios and confidence intervals for mortality rates on or after 2000 compared with 1995 and before, using a linear combination of variables from the Poisson regression analysis. Mortality from cancer, ischaemic heart disease, and cerebrovascular disease among clerical and sales workers decreased significantly by over 20% on or after 2000 compared with 1995 or before. However, mortality from cancer and ischaemic heart disease among agricultural workers, and from suicide among professional and service workers increased significantly after 2000.

**Table 4:** Relative change in occupation-specific mortality on or after 2000 compared with 1995 or before among female workers

Cause of death and occupational category	Rate ratio^a^	95% confidence interval	*p *value
*All cancers*			
Professional	0.97	(0.92–1.03)	0.323
Clerk	0.70	(0.67–0.74)	< 0.001
Sales	0.80	(0.76–0.84)	< 0.001
Service	0.94	(0.90–0.99)	0.024
Agriculture	1.17	(1.10–1.25)	< 0.001
Manufacturing	0.81	(0.77–0.86)	< 0.001
Other	1.59	(1.49–1.70)	< 0.001
*Ischaemic heart disease*			
Professional	1.16	(0.91–1.48)	0.235
Clerk	0.75	(0.60–0.93)	0.009
Sales	0.82	(0.67–1.01)	0.057
Service	0.91	(0.76–1.09)	0.308
Agriculture	1.33	(1.06–1.66)	0.013
Manufacturing	0.93	(0.76–1.13)	0.441
Other	1.66	(1.27–2.16)	< 0.001
*Cerebrovascular disease*			
Professional	1.10	(0.98–1.24)	0.107
Clerk	0.88	(0.79–0.97)	0.015
Sales	0.83	(0.75–0.92)	< 0.001
Service	1.01	(0.92–1.10)	0.911
Agriculture	1.11	(0.98–1.25)	0.111
Manufacturing	0.91	(0.82–1.01)	0.068
Other	1.52	(1.31–1.75)	< 0.001
*Suicide*			
Professional	1.45	(1.28–1.66)	< 0.001
Clerk	1.03	(0.91–1.16)	0.633
Sales	1.00	(0.88–1.15)	0.975
Service	1.35	(1.20–1.53)	< 0.001
Agriculture	1.16	(0.99–1.34)	0.060
Manufacturing	1.00	(0.88–1.14)	0.956
Other	1.99	(1.66–2.38)	< 0.001

^a^Rate ratio on or after 2000 compared to 1995 or before obtained from Poisson regression analysis adjusting for year, age category, step variable (mortality before or after 2000), occupation and interaction of step variable and occupation

## Discussion

This study found a decreasing trend in mortality from all cancer deaths combined, ischaemic heart disease, cerebrovascular disease, and suicide among women in all occupational groups from 1980 to 2015. Mortality risk among agricultural workers was higher, while among manufacturing and clerical workers were lower than among professionals for all four causes of deaths. The absolute decline in mortality rates from all cancers and cerebrovascular disease was higher in 2000–2015 than 1980–1995. After 2000, clerks and sales workers had a decreasing trend in mortality from all causes except suicide. However, the rates of mortality from all four causes among agriculture workers increased slightly after 2000.

Previous research has identified inequalities in Japanese women’s mortality profiles. The risk of stroke is higher, while the risk of ischaemic heart disease is lower among Asians [20]. A study found a higher incidence of stroke among Japanese women with Junior High School- and College-level education than women with High School-level education, demonstrating a U-shaped association [21]. The risk factors for ischaemic heart disease have changed in women as they have in the population as a whole, and the downward trends in mortality are consistent with these gains [22]. The rate of smoking, which is a major risk factor for cerebral infarction [23], is generally low among Japanese women of all ages [20]. During the 1980s and 1990s, public health endeavours focused on stroke and other cerebrovascular diseases. The prevalence of hypertension decreased with improvements in lifestyle, reduction in salt intake among women and expanding coverage of medications for hypertension from 1980 to 2013 [24, 25]

The cancer mortality rates across all occupational categories of Japanese women, which have been declining since the early 1980s, decreased further after the Cancer Control Act was approved in 2006 [26]. With advances in treatment and improved prognosis for breast cancer, which is the most common cancer in Japanese women, the survival rate has improved [27], leading to a significant reduction in mortality rates. There are socio-economic inequalities in cancer incidence among female workers in Japan [28], but public health efforts have resulted in population-wide changes, leading to a reduction in cancer mortality rates. However, cervical screening rates in Japanese women remain low and vaccination programs against human papillomavirus have stalled [29], so gains in this other common cause of cancer mortality are threatened. Reinstatement of the human papillomavirus vaccination program and enhanced efforts on cervical screening are needed to continue to improve cancer mortality rates in the future.

Japan has one of the highest suicide rates in the world. The rate, however, has been decreasing in recent years with 18.9 per 100,000 population in 2015 [30, 31]. We found stable suicide rates across all occupational categories except professional and service workers. Previous studies reported that nurses, doctors, dentists, pharmacists and police officers are at higher risk of suicide [32, 33]. The impact of socioeconomic factors on suicide in greater in Japan compared to other Organisation for Economic Co-operation and Development countries [34, 35]. Suicide has been identified as a potential contributor to the rise in inequalities in mortality because studies suggest associations between increasing suicide rates and the global financial crisis [7]. Recent work on excess suicide mortality among Japanese women during the coronavirus pandemic suggests that Japanese women are more vulnerable to socio-economic disruption than men, and extra prevention activities may be required in times of economic or social disturbance [36]. This highlights the importance of suicide prevention to reduce inequalities in mortality, especially during turbulent economic conditions.

We found a decrease in both absolute and relative mortality across all occupations for 1980–1995 and 2000–2015. Similarly, mortality from all cancers, ischaemic heart disease and cerebrovascular disease decreased significantly after 2000 among clerical and sales workers compared with before 1995. Studies suggest that women in lower socio-economic classes in Japan are more likely to take risky jobs, resulting in higher mortality rates [37]. This potentially explains the higher rates of mortality among agricultural workers from all four causes. However, these higher rates could also be a result of the rapidly ageing agricultural workforce in Japan [38]. A large proportion of women worked as clerks, the jobs that are often considered at least risk during economic recessions. Similarly, women in higher socio-economic positions may have access to extensive resources as a result of their widespread networks [39], leading to reduced mortality rates than in other occupational categories.

Our study has several limitations. There is a possibility that the distribution of population is different across occupations as we used data from two different sources for information related to deaths and population. Thus, this study is attributable to numerator-denominator bias. However, the result from the sensitivity analysis in a study by Wada et al. showed a minimal effect of the numerator-denominator bias [7]. The occupation was based on subjective information from the family of deceased women potentially leading to recall bias. As almost 44% of Japanese women were part-time or irregular workers [14], the deceased women’s occupation may not have been declared by the family member. This information could therefore be misleading, because it may reflect long-term occupation rather than any occupation that the deceased woman was engaged in immediately before her death. Some occupations may have been misclassified as unemployed or others if the person quit her job because of serious illness. We used the Japan Standard Occupational Classification to categorise occupations. However, it has been revised several times over the years, which may have resulted in bias in our findings. Agricultural workers were the smallest category throughout the study period, and this may be reflected in the calculated age-standardised rates and, hence, trends.

## Conclusions

In conclusion, mortality rates have decreased across all occupational categories over the years, reducing the prevailing inequalities in mortality. However, attention should now be given to agricultural workers to further reduce the mortality gap. Continuous monitoring of mortality trends is essential to ensure better health and wellbeing in Japan.

## Supplementary Information

**Additional file 1.** **Table A1:** Rate ratios of mortality rates for the leading causes of death Description of table: Result of Poisson regression analysis.

## Data Availability

The data that support the findings of this study are available from the Japanese Ministry of Health, Labour and Welfare but restrictions apply to the availability of these data and so are not publicly available.
