# Supplementary material for: Changes in cause-specific mortality trends across occupations in working-age Japanese women from 1980 to 2015: a cross-sectional analysis
Source: BMC Womens Health. 2022 Feb 22;22:44. doi: 10.1186/s12905-022-01621-4 (PMC8861597; doi:10.1186/s12905-022-01621-4)
Supplement: Supplementary file 1 — Additional file 1. Table A1: Rate ratios of mortality rates for the leading causes of death Description of table: Result of Poisson regression analysis. [file 12905_2022_1621_MOESM1_ESM.pdf]

Additional file

Table A1: Rate ratios of mortality rates for the leading causes of death

Cause of death and variable	Rate ratio[a]	95% Confidence Interval	p-value
**All cancers**			
Time	0.978[b]	(0.976–0.979)	<0.01
Age category			
35-44	3.097	(2.954–3.248)	<0.01
45-54	7.520	(7.193–7.862)	<0.01
55-64	14.145	(13.531–14.788)	<0.01
Occupation			
Clerk	0.687	(0.657–0.718)	<0.01
Sales	0.927	(0.887–0.969)	<0.01
Service	0.804	(0.768–0.842)	<0.01
Agriculture	1.049	(1.004–1.095)	<0.05
Manufacturing	0.460	(0.440–0.480)	<0.01
Others	2.971	(2.814–3.137)	<0.01
After 2000	0.973	(0.921–1.028)	0.323
Occupation / After 2000			
Clerk X After 2000	0.723	(0.680–0.770)	<0.01
Sales X After 2000	0.822	(0.771–0.877)	<0.01
Service X After 2000	0.969	(0.910–1.031)	0.317
Agriculture X After 2000	1.206	(1.123–1.294)	<0.01
Manufacturing X After 2000	0.833	(0.780–0.889)	<0.01
Others X After 2000	1.636	(1.518–1.763)	<0.01
**Ischaemic heart disease**			
Time	0.970[b]	(0.964–0.976)	<0.01
Age category			
35-44	2.133	(1.625–2.800)	<0.01
45-54	5.968	(4.618–7.712)	<0.01
55-64	17.900	(13.885–23.076)	<0.01
Occupation			
Clerk	0.712	(0.577–0.877)	<0.01
Sales	1.422	(1.171–1.726)	<0.01
Service	1.529	(1.258–1.859)	<0.01
Agriculture	1.488	(1.229–1.801)	<0.01
Manufacturing	0.673	(0.553–0.820)	<0.01
Others	4.060	(3.201–5.148)	<0.01
After 2000	1.160	(0.908–1.483)	0.235
Occupation / After 2000			
Clerk X After 2000	0.644	(0.482–0.859)	<0.01
Sales X After 2000	0.708	(0.539–0.930)	<0.05
Service X After 2000	0.784	(0.605–1.015)	0.065
Agriculture X After 2000	1.143	(0.857–1.524)	0.362
Manufacturing X After 2000	0.797	(0.606–1.047)	0.103
Others X After 2000	1.427	(1.034–1.970)	<0.05
**Cerebrovascular disease**			
Time	0.957[b]	(0.954–0.960)	<0.01
Age category			

35-44	3.778	(3.380–4.222)	<0.01
45-54	11.255	(10.134–12.500)	<0.01
55-64	21.135	(19.031–23.473)	<0.01
Occupation			
Clerk	0.702	(0.639–0.770)	<0.01
Sales	1.366	(1.252–1.490)	<0.01
Service	1.439	(1.319–1.571)	<0.01
Agriculture	1.519	(1.394–1.655)	<0.01
Manufacturing	0.661	(0.605–0.721)	<0.01
Others	3.210	(2.871–3.589)	<0.01
After 2000	1.103	(0.979–1.242)	0.107
Occupation / After 2000			
Clerk X After 2000	0.795	(0.693–0.913)	<0.01
Sales X After 2000	0.749	(0.654–0.857)	<0.01
Service X After 2000	0.912	(0.803–1.034)	0.151
Agriculture X After 2000	1.002	(0.862–1.165)	0.978
Manufacturing X After 2000	0.824	(0.720–0.944)	<0.01
Others X After 2000	1.374	(1.160–1.628)	<0.01
**Suicide**			
Time	0.977[b]	(0.973–0.981)	<0.01
Age category			
35-44	0.910	(0.856–0.967)	<0.01
45-54	1.108	(1.045–1.174)	<0.01
55-64	1.129	(1.059–1.203)	<0.01
Occupation			
Clerk	0.712	(0.638–0.794)	<0.01
Sales	1.118	(0.999–1.250)	0.052
Service	1.292	(1.156–1.445)	<0.01
Agriculture	2.482	(2.235–2.757)	<0.01
Manufacturing	0.714	(0.641–0.796)	<0.01
Others	3.537	(3.050–4.103)	<0.01
After 2000	1.453	(1.276–1.656)	<0.01
Occupation / After 2000			
Clerk X After 2000	0.709	(0.611–0.821)	<0.01
Sales X After 2000	0.690	(0.589–0.808)	<0.01
Service X After 2000	0.931	(0.806–1.076)	0.335
Agriculture X After 2000	0.795	(0.670–0.943)	<0.01
Manufacturing X After 2000	0.691	(0.591–0.806)	<0.01
Others X After 2000	1.366	(1.119–1.668)	<0.01

[a]Result of Poisson regression analysis obtained by adjusting for year, age category, step variable indicating mortality before or after 2000, occupation and an interaction of step variable and occupation; [b]per five year
